# Prognostic factors in prostate cancer

**DOI:** 10.1186/1746-1596-1-4

**Published:** 2006-04-03

**Authors:** A Buhmeida, S Pyrhönen, M Laato, Y Collan

**Affiliations:** 1 Departments of Oncology and Radiotherapy, Turku University Hospital, Turku, Finland; 2 Departments of Pathology, Turku University Hospital, Turku, Finland; 3 Departments of Surgery, Turku University Hospital, Turku, Finland

## Abstract

Prognostic factors in organ confined prostate cancer will reflect survival after surgical radical prostatectomy. Gleason score, tumour volume, surgical margins and Ki-67 index have the most significant prognosticators. Also the origins from the transitional zone, p53 status in cancer tissue, stage, and aneuploidy have shown prognostic significance. **Progression-associated features **include Gleason score, stage, and capsular invasion, but PSA is also highly significant. Progression can also be predicted with biological markers (E-cadherin, microvessel density, and aneuploidy) with high level of significance. Other prognostic features of clinical or PSA-associated progression include age, IGF-1, p27, and Ki-67. In patients who were treated with radiotherapy the survival was potentially predictable with age, race and p53, but available research on other markers is limited. The most significant published **survival-associated prognosticators **of prostate cancer with extension outside prostate are microvessel density and total blood PSA. However, survival can potentially be predicted by other markers like androgen receptor, and Ki-67-positive cell fraction. In advanced prostate cancer nuclear morphometry and Gleason score are the most highly significant progression-associated prognosticators. In conclusion, Gleason score, capsular invasion, blood PSA, stage, and aneuploidy are the best markers of progression in organ confined disease. Other biological markers are less important. In advanced disease Gleason score and nuclear morphometry can be used as predictors of progression. Compound prognostic factors based on combinations of single prognosticators, or on gene expression profiles (tested by DNA arrays) are promising, but clinically relevant data is still lacking.

## Introduction

Prostate cancer is the most common malignancy in men and the second leading cause of cancer death in the Western world [1-3]. Today, more patients with prostate cancer are being diagnosed in early stages of the disease than used to be the case 10 years ago. The increasing incidence may be due to increased PSA-measurements and other diagnostic efforts. However, this review does not handle the associated differential diagnosis. Also, the biological heterogeneity that characterizes this disease causes decision issues unique to prostate cancer. Low-grade cancer diagnosed late in life may have no impact on the quality or length of life. A younger man with a high-grade lesion may have advanced disease and die within a couple of years. Biological distinction of such patients should have a high priority in continuing research.

Although prostate cancer is very prevalent among men, relatively little is known about the molecular mechanisms involved in the development and progression of the disease [4-7]. The lack of knowledge on the biology of prostate cancer has resulted in numerous controversies on the clinical management of the early stages and on the utility of population screening [8].

One of the aims of molecular genetics is to reveal the genetic alterations and genes that are involved in disease processes. Molecular pathogenesis of the prostate cancer is poorly understood. Over the past 10 years, chromosomal aberrations in prostate cancer have been studied with several techniques, such as loss of heterozygosity (LOH), fluoresence in situ hybridization (FISH), comparative genomic hybridization (CGH), suppression substractive hybridization (SSH) and cDNA array hybridization. FISH has been used to identify the target genes for some of these chromosomal alterations [9]. These chromosomal alterations are most likely to harbour the genes critical for the progression of prostate cancer [10].

After the diagnosis is established, the physician wants to determine whether the lesion is confined to the prostate gland (and is hence potentially curable), or whether cancer has spread beyond the prostate and is incurable. At the present time, the successful radical treatment of prostate cancer is limited to the patients with organ-confined disease.

On the other hand, there are also latent prostate cancers. Most patients with latent prostate cancer die with, rather than of, prostate cancer. Against this background we should concentrate in finding objective criteria for distinguishing between clinically significant and clinically insignificant cancers, usually falling into the category of latent prostate cancer [11]. In other words, it is becoming increasingly important to find factors, which could predict which patients have tumours with aggressive invasive potential to spread outside the prostate [12]. Various types of clinical and pathological information may contribute in building decision making systems or tools for this purpose. Clinicians treating prostate cancer patients may potentially use these tools.

The management could be in the form of early detection and identification of prognostic factors, which help in forecasting the outcome in an individual case. Perfect forecasting could help in selecting the treatment mode that would be most appropriate for the treatment of an individual patient. So patients with favourable outcome – if identified – would not need crippling therapy whereas patients with a high risk of early metastasis or death would be placed in the group of more intensive treatment and surveillance follow up. This is also important after surgical radical prostatectomy, which offers lot of material for analysis and creates a challenge for the pathologists [13]

Unfortunately, general reliable forecasting of the outcome is still not possible, and efforts to identify prostate cancer prognosticators must continue [14]. So, it is important to answer the question: What are the histolgical, cell biological and molecular features which could differentiate between aggressive and non-aggressive types of prostate cancer?

The intention of this review is to shed light on the above questions by reviewing the most widely studied prognostic factors to help the clinicians to create practical prognostic models that can potentially also help in individualization of the treatment. Some of these important prognostic factors are summarized in Table 1.

**Table 1:** Prognostic factors associated with prostate cancer after different types of treatment and according to the level of extension. Patients with organ confined disease treated with radical prostatectomy (surgical or radiated), and patients with more extensive disease are presented separately. The database applied was PubMed, the presented papers were published during years 1989 – 2005.

**Organ confined disease, radical prostatectomy (surgical)**					
***Survival-associated prognosticators***			***Progression-associated prognosticators***		
**Prognosticator**	**p value**	**Reference**	**Prognosticator**	**p value**	**Reference**
Tumour volume	< 0.009	Salomon et al.	Age	< 0.01	Obek et al.
Gleason score	< 0.0002	Hoznek et al.	Gleason score	< 0.0001	Epstein et al.
Surgical margin	< 0.009	Hoznek et al.	Capsular invasion	< 0.001	Wheeler et al.
Tranzitional zone	< 0.04	Augustin et al.	PSA*	< 0.001	Salomon et al
p53	< 0.011	Kuczyk et al.	Surgical margin	< 0.075	Bloom et al.
Stage	< 0.02	Kuczyk et al.	E-cadherin	< 0.005	Umbas et al.
p27	< 0.01	Yang et al.	IGF-1**	< 0.05	Yu et al.
Aneuploidy	< 0.02	Zincke et al.	Stage	< 0.001	D'Amico et al.
Ki-67	< 0.001	Bettencourt et al.	MVD***	< 0.007	Halvorsin et al.
cDNA microarray	< 0.01	Susan et al.	p27	< 0.008	Yang et al.
			Aneuploidy	< 0.0001	Zincke et al.
			Aneuploidy	< 0.001	Ross et al.
			Ki-67	< 0.02	Bubendorf et al.
			MUC1	< 0.003	Lapointe et al.
**Organ confined disease, radical prostatectomy (radiation)**					

***Survival-associated prognosticators***			***Progression-associated prognosticators***		
**Prognosticator**	**p value**	**Reference**	**Prognosticator**	**p value**	**Reference**
Age	< 0.02	Austin el al.			
			Age	< 0.01	Neulander et al
Race	< 0.02	Austin et al.			
			Gleason score	< 0.001	Kupelian et al.
p53	< 0.02	Grignon et al.			
			Radiation dose	< 0.001	Kupelian et al.
**Prostate cancer with extension outside the prostate (advanced disease)**					

***Survival-associated prognosticators***			***Progression-associated prognosticators***		
**Prognosticator**	**p value**	**Reference**	**Prognosticator**	**p value**	**Reference**
AR****	< 0.01	Segawa et al.	Nuclear morphometry	< 0.01	Partin et al.
AR	< 0.02	Miyoshi et al.	Nuclear morphometry	< 0.0003	Vesalainen et al.
Total-PSA	< 0.001	Bjork et al.	Gleason score	< 0.0001	Vesalainen et al.
MVD	< 0.0001	Borre et al.	Gleason score	< 0.007	Shurbaji et al.
Ki-67	< 0.02	Aaltomaa et al.	AR	< 0.03	Sadi et al.
			p53	< 0.018	Bauer et al.
			bcl-2	< 0.004	Bauer et al.

* Prostate Specific Antigen, ** Insulin-like Growth Factor, *** Microvessel density, **** Androgen Receptor

### Clinico-pathological prognostic factors

Clinical prognostic factors are those that can be assessed through physical examination: blood tests, radiological evaluation, and microscopy of biopsy material. Clinical factors are important because they allow the cancer to be characterized before a definitive treatment decision is made. In this review article, pathological prognostic factors are those that require examination, removal, and evaluation of the entire prostate.

#### Age of patient

The role of the age of the patient per se as a significant prognostic factor in prostate cancer is controversial [15,16]. 567 patients completing external beam radiotherapy were examined by Herold et al [17]. In addition to other factors, age of the patients greater than 65 years was a significant predictor of distant metastases at 5 years. They concluded that men over the age of 65 years were more likely to experience distant failure after radical radiation therapy than were younger men. Obek et al [18] also suggested that young age per se might be an independent favourable prognostic factor for disease recurrence after surgical radical prostatectomy. Also Freedland et al [19] found that young men had more favourable outcomes after surgical radical prostatectomy (RP) than older men, which made younger men suitable subjects in screening.

#### Volume

Although tumour volume is an important factor in predicting prognosis in carcinoma of the prostate, direct and accurate estimation of tumour volume is not practical clinically. This is because the tumour may not always be palpable, and when palpable the volume cannot be evaluated in 3 dimensions [20]. Transrectal ultrasound (TRUS) used as a tool for estimating the tumour volume either directly (Terris et al 1992) [21] or as a guide for core biopsies [22,23] has only limited ability to estimate prostate cancer volume. 176 radical prostatectomy specimens were studied with respect to cancer volume by McNeal et al [24]. They found that the extent of capsular penetration, cancer volume, and positive nodes were strongly inter-correlated. Twelve cubic centimetres was the critical volume. Higher volumes were usually associated with extensive capsular penetration, positive surgical margins, and/or positive nodes.

To determine which of these three variables provided independent prediction of prognosis Epstein et al [25] evaluated 185 men following surgical radical prostatectomy for stage T2 disease. The patients were re-evaluated at 5 years after prostatectomy and grouped into 2 groups: those free of disease, and those who experienced progression. The study accepted only cases with negative pelvic lymph nodes and with negative seminal vesicles at the time of the diagnosis. At five years following radical prostatectomy, 58 men (31%) had experienced progression. Gleason grade, surgical margins, and tumour volume each where highly correlated with progression. In stepwise regression analysis, tumour volume did not provide independent prognostic information beyond that provided by Gleason score and the status of surgical margins.

The study by Bostwick et al [26] for evaluating the utility of tumour volume in predicting progression of early prostate cancer revealed that tumour volume was a significant predictor of cancer progression. They found that there was a 10% probability of capsular invasion in tumours measuring about 0.5 cm^3^, 10 % probability of seminal vesicle invasion in tumours measuring about 4.0 cm^3^, and 10 % probability of distant metastases in tumours measuring about 5.0 cm^3^. As described by McNeal et al [27] loss of differentiation and potential to give rise to metastases were strongly correlated with tumour volume. In contrast to the previous results, Salomon et al [28] showed that tumour volume does not provide additional information to predict prostate cancer progression after radical prostatectomy. The same was concluded by Kikuchi et al [29]. Simply because tumour volume does not give additional prognostic value to the Gleason score, many centers do not consider it valuable in clinical practice [13]. However, if better prognosticators are not available, the volume is still a valuable guide in prognostication.

#### Grading

All existing grading systems successfully identify well-differentiated cancer, which progresses slowly, and poorly differentiated cancer, which progresses rapidly, but they are less successful in subdividing moderately differentiated cancers, which have an intermediate malignant potential [30]. The histological Gleason score of the adenocarcinoma of the prostate is a good and an established prognostic indicator.

Dr Donald F Gleason [31] and members of the Veterans Administration Cooperative Urological Research Group devised the Gleason grading method in the 1960s and 1970s. This grading system is based entirely on the histological pattern of differentiation and arrangement of carcinoma cells and cell groups in H&E-stained sections. Five basic patterns (scored 1–5) are used to generate a histological sum score (summed from scores of two most dominant patterns), which can range from 2 to 10 [32]. Gleason grading of the cancer is the most widely used, and accepted histopathological method for providing information about the prognosis of prostate cancer. Univariate and multivariate analyses of prognosis in prostate cancer almost always identify Gleason grade as one of the most significant predictors of patient outcome [33]. A 3-grade grading system is also in use, but no longer recommended [13]. Also, models have been developed which allow for pre-treatment prediction of pathologic stage on the basis of needle biopsy Gleason grade, total serum prostate-specific antigen level, and clinical stage [34]. It is of considerable interest to know how accurate the needle biopsy Gleason score is in relation to Gleason score obtained from the radical prostatectomy samples [35]. Over half of the patients are under- or over-graded by needle biopsy. Clinicians should be aware of this potential inaccuracy when using Gleason grading in decision-making [36]. Koksal et al [37] found that grading error was greatest in well-differentiated tumours and true sum scores between 2–4. The grading error decreased with increasing Gleason score. The same was concluded by Shen et al [38]. Tumours with combined Gleason scores of 2–4 are not commonly present in needle biopsy material, since palpable tumours are usually of a higher grade [39].

Two important studies [40,41] have demonstrated a good correlation between the prognosis of prostate cancer and combined Gleason scores. Even when a high-grade tumour is organ confined, it is associated with a relatively unfavourable short-term outcome that is not predictable on the basis of either preoperative clinicopathologic data or postoperative pathologic information obtained from the radical prostatectomy specimen [42]. The study by Cheng et al [43] showed that the combined percentage of Gleason patterns 4 and 5 is the best predictor of progression after radical prostatectomy. This is why the percentage of patterns 4 and 5 should today be reported in histopathological evaluation. With Partin coefficient tables it is possible to calculate risks for recurrence [44]

#### Extracapsular extension

In rectal examination the incidence of capsular penetration in palpable clinically confined tumours is 20–38% or 40–66% (unilateral or bilateral tumours, respectively) [45,46]. The study of 196 tumours by Epstein et al [47] demonstrated that tumours with more extensive capsular penetration had a higher risk of progression than those showing focal capsular penetrations. One-hundred thirty (130) patients with follow up of more than 10 years after radical prostatectomy were histologically restaged by Theiss et al [48]. They found that in contrast to capsular invasion as such, capsular penetration is an indicator of poor prognosis. Capsular penetration was associated with higher progression rate and reduced survival. They recommended that tumours with capsular invasion and those with capsular penetration should be distinguished. Ohori et al [49] found that the probability of progression-free survival at 7 years was 65% for patients with extracapsular extension and positive margins and a Gleason score of 6 or less, and 40% for patients with extracapsular extension and positive surgical margins and a Gleason score of 7 or more. To assess the relationship between the level and extent of prostatic capsular invasion by cancer, the clinical and pathological features, and prognosis of early state prostate cancer Wheeler et al [50] used multivariate analysis. They found that the level of capsular invasion was an independent prognostic factor and a strong association between the level of invasion of cancer into or through the prostatic capsule and the volume, grade, pathological stage, and the rate of recurrence after radical prostatectomy. Also they concluded that sub-classification of patients according to the levels of prostatic capsular invasion provides valuable prognostic information.

#### Seminal vesicle invasion

In most recent studies, seminal vesicle invasion (SVI) is a poor prognostic parameter [51,52], with biochemical progression-free rates ranging from 5–60%. The differences may be related to the definition of the seminal vesicle invasion. Some authors consider an intraprostatic portion of the seminal vesicle as true seminal vesicle, and as such consider its involvement by cancer as seminal vesicle invasion. Others call any seminal vesicle as extracapsular extension [53]. Some studies do not make any distinction between the seminal vesicles and the ejaculatory duct complex.

Seminal vesicle invasion is associated with high PSA failure rates (PSA levels not changed to normal) after radical prostatectomy, and subsequent distant metastases [54]. Debras et al, [55] evaluated the prognostic significance of SVI in radical prostatectomy specimens according to proximal or distal site of invasion. They concluded that the prognostic significance of SVI is not constant and depends on the site of invasion, in which patients with invasion extending to the free part of the seminal vesicles have poorer prognosis than those patients with invasion only limited to the proximal part of the seminal vesicles.

115 cases of established capsular penetration, 16 of peri-seminal vesicle invasion, and 45 of seminal vesicle invasion in-patients without lymph node metastases were evaluated by Epstein et al (1993c). They concluded that patients with SVI had a significantly worse prognosis than those with capsular penetration, and peri-seminal vesicle invasion was associated with an intermediate risk of progression. The results of Freedland et al [56] revealed that patients with SVI had significantly higher PSA values, higher clinical stage, higher grade tumours, and were more likely to have concomitant extracapsular extension or a positive surgical margin. The study also identified a subset of men with low-grade disease, negative surgical margins, and older age, who – despite SVI – had an extremely favourable clinical course. The study concluded that SVI does not consistently suggest an unfavourable prognosis.

#### Zone of origin

The development of zonal anatomy of the prostate reported by McNeal [57,58] allows the assignment of the zone of origin to individual prostate cancer foci. Cancer foci detected incidentally in tissue removed by transurethral resection of prostate (TURP) (stage A) are predominantly of transitional zone (TZ), while clinically palpable (stage B) cancers are predominantly of peripheral zone (PZ) origin [59]. McNeal identified 68% of small prostate cancers as originating from the peripheral zone (PZ), 24% from the transitional zone (TZ), and 8% from the central zone (CZ).

To determine the characteristics of transition zone and peripheral zone prostate cancer, Greene et al [60] examined a series of 42 stage A and 54-stage B radical prostatectomy specimens. They paid particular attention to the number of separate foci of cancer, zone of origin, volume and grade of foci, presence of severe intraductal dysplasia, extra-capsular extension, and seminal vesicle invasion associated with cancer in each zone. They found that there were fundamental differences between transitional zone (TZ) and peripheral zone (PZ) cancers. Cancer that arises in the transitional zone is associated with more favourable pathological features and may have less malignant potential than tumours that arise in the peripheral zone. The results of Augustin et al [61] showed that patients with tumours including 70% or more of the cancer volume in the TZ had a significantly higher rate of biochemical cure than those with 30% or less. Jack et al [62] revealed that transitional zone tumours were favourable with higher rate of organ confined and lower grade tumours. They concluded that if transitional zone tumours prove to be biologically distinct, improved strategies to identify these lesions preoperatively might result in more conservative treatment recommendations.

#### Heterogeneity and multicentricity

As early as 1935, Moore had recognized that prostatic carcinomas are often multifocal [63]. Prostatic carcinoma is characteristically multifocal with as many as 5 or 6 tumours occurring in a single prostate [64]. A great challenge for diagnostic pathologists was the characteristic heterogeneous appearance of prostatic carcinoma. The availability of radical prostatectomy specimens has provided the opportunity to examine the interrelationships of histological heterogeneity and multicentricity in individual specimens [65]. The influence of grade heterogeneity and tumour multifocality on the ability to predict the prognosis of patients with prostate cancer is profound. The multifocal and heterogeneous nature of prostate makes it difficult to obtain representative biopsy samples from the tumours [66]. Hammerer and associates [67] considered the number of biopsies positive for cancer as quantitative measure of tumour multicentricity. The data of Djavan et al [68] suggested that multifocal prostate cancer is associated with higher grade, stage, and recurrence rate than unifocal prostate cancer.

#### Morphometric features

In 1982 Diamond and associates introduced nuclear morphometry to aid in prediction of prognosis among patients with prostate cancer [69,70]. He and his colleagues observed that nuclear roundness was very useful in separating long survivors among stage B patients from those who develop metastasis. They observed no overlap in nuclear roundness between the two groups. Since then, many histological studies [71-75] have used nuclear morphometry to predict prognosis in patients with prostate cancer. Eichenberger and associates [73] calculated 12 shape descriptors including nuclear roundness, ellipticity factors, and concavity factors. They used discriminate analysis to select the major morphometric parameters which best distinguished patients with good or poor prognosis. Elliptical shape measurement was found to be the best in this respect.

To evaluate critically the usefulness of nuclear morphometry for prediction of prognosis, Partin et al [75] developed a morphometric evaluation system called Hopkin's Morphometry System, and produced and compared 15 different shape descriptors in stage A2 prostate cancer. These were analysed by 17 different statistical tests. The best separation was provided by the lower quartile analysis of the ellipticity shape descriptor (p < 0.01). These studied revealed that elliptical shape of the nuclei is very important as prognostic factor.

Variance of nuclear roundness combined with clinical stage, Gleason score, and age produced a prognostic score capable of stratifying patients with clinically localized cancers into three groups with different disease free survival [76].

Aragona et al [77] concentrated on the role of size features. They found that accurate prediction for well-differentiated adenocarcinoma was attained using mean nuclear area (> 28 μm^2^, mean nuclear diameter (> 5 μm) and the presence of more than 5% of cells with a nuclear diameter greater than (6.16 μm).

A series of 325 patients with prostatic adenocarcinoma were followed-up for over 13 years by Vesalainen et al [78]. The patients were subjected to histomorphometric analysis for the following prognostic factors: the Gleason score and 10 nuclear morphometric factors (mean nuclear area, nuclear perimeter, and shortest and longest nuclear factors, form factors and their SDs). They found that in T1-2M0 tumours, all of these parameters were significant prognostic factors. The results of study by Martinez-Jabaloyas et al [79] revealed that mean nuclear area and other factors proved to have prognostic value in the univariate analysis and concluded [80] that nuclear morphometry in the primitive tumour provides independent prognostic information in survival analysis for patients with metastatic prostate cancer. The combined evaluation of high nuclear morphology, ploidy and cell survival parameters such as Bcl-2 expression might better identify patients with poor prognosis among early stage prostate carcinomas diagnosed by FNA biopsies [81].

Beside to the prognostic and predictive power of morphometry, Buhmeida et al [82] revealed that the nuclear size features are useful in distinguishing between different atypia groups of prostate gland in fine needle aspiration biopsies, particularly if the sample-associated means of the size features (area, diameter, perimeter, short and long axes) are used for the interpretation of data. The study suggested that if the upper range limit of sample-associated mean areas of nuclei is below 27 μm^2^, it is most probable that we are dealing with benign cells. If the upper range limit is above 39 μm^2^, it is possible that there are malignant cells in the sample. However, values above 52 μm^2 ^represent malignant samples with certainty. Further studies will be necessary for associating nuclear size features with Gleason grades.

### Biological prognostic factors

#### E-cadherin

Normally functioning cell-cell adhesion plays an important role in the maintenance of tissue architecture and cohesion. E-cadherin is an important adhesion molecule in epithelial cells. E-cadherin expression has been proposed for predicting prognosis in prostate adenocarcinoma. A study of E-cadherin levels by immunohistochemistry in nonmalignant and malignant specimens of human prostatic tissue revealed that almost 50% of tumours examined had reduced levels of this protein, and in some tumours E-cadherin was absent altogether when compared to non-malignant prostate, which uniformly stained strongly positive [83]. To determine the potential prognostic significance of the findings, prostate cancer specimens from 89 patients were evaluated immunohistochemically using specific antibodies raised against E-cadherin [84]. The results were related to histological grade, tumour stage, presence of metastasis, and survival. Patients showing low immunohistochemical expression of E-cadherin have on average shorter survival than patients with high immunohistochemical expression.

Because mutational inactivation of alpha-catenin can be the cause of the impaired E-cadherin function, Umbas et al [85] studied the relationship between E-cadherin and alpha-catenin expression. The results suggest that loss of alpha-catenin expression could be one of the mechanisms responsible for the loss of E-cadherin mediated cell-cell adhesion in human prostate cancer and might in some cases provide prognostic information. The same was concluded by Aaltomaa et al [86] who studied the expression of alpha-catenin in locally advanced prostate cancer. They found that alpha-catenin had prognostic significance in the early phases of cancer progression. Low alpha-catenin expression was related to worse prognosis than high alpha-catenin expression. De-Marzo et al [87] correlated the down-regulation of E-cadherin and pathologic stage at radical prostatectomy. In univariate analysis they found that reduced levels of E-cadherin correlated with advanced Gleason score (p = 0.003) and advanced pathologic stage (p = 0.008). In multivariate analysis, E-cadherin, preoperative PSA, and Gleason score all contributed independently to the prediction of high stage disease (p < 0.001). They concluded that a prospective study on E-cadherin is warranted. The study should evaluate E-cadherin as a potential biomarker of disease progression in patients with clinically organ-confined prostate cancer who undergo radical prostatectomy. Moderate or strong expression of a transcriptional repressor EZH2 (enhancer of zestor homolog2) coupled with at most moderate expression of E-cadherin was the biomarker combination that was most strongly associated with recurrence of prostate cancer [88]. In the clinical situation low E-cadherin immunostaining suggested clinical recurrence. But to what extent and at what level of accuracy the status of an individual patient can be predicted should be evaluated in further studies.

#### Insulin-like growth factor (IGF)

The insulin-like growth factor (IGF) system is composed of two ligands (IGF-I and IGF-II), two receptors (IGFR-I and IGFR-II) and six binding proteins (IGFBP 1 to 6). Mita et al [89] found that IGF-II and IGFBP2 play a role in prostate cancer progression and their increased expression is a prognostic indicator in hormone- treated prostate cancer patients. The results of the study by Figueroa and co-workers [90] indicate that the higher expression of IGFBPs in human prostate cancer correlates with the Gleason score, and the expression of certain IGFBPs may be used as markers of aggressive clinical behaviour. After studying changes in IGFBP2 and IGFBP3 levels in serial postoperative serum samples from prostate cancer in patients with and without relapse, Yu et al [91] suggested that IGFBP2 may play a role in the progression of prostate cancer, but that serum levels of IGF-I and IGFBP3 have no predictive value in the progression of prostate cancer. However, the high preoperative circulating plasma insulin-like growth factor (IGF-I) levels have been correlated with increased risk of prostate cancer [92-94].

#### Androgen receptors (AR)

The androgen receptor (AR) is a nuclear transcription factor that binds male sex steroids and mediates the biological effects of these hormones in the target cells by activating transcription of androgen-dependent genes. The AR gene is localized on chromosome X and it contains a series of CAG trinucleotide repeats. The length of CAG repeats varies among individuals and this polymorphism is believed to be related to the transcriptional activity of AR. Fewer CAG repeats are associated with increased risk of developing tumour as well as more aggressive forms of prostate cancer and breast cancer of women [95].

Withdrawal of androgens or peripheral blockade of androgen action is a critical therapeutic option in the treatment of advanced prostate cancer. However, after initial regression, many prostate cancers become hormone refractory and progress further with eventually fatal outcome. A large number of different molecular mechanisms may be responsible for the development of hormone-refractory recurrent tumours. Many of these involve the AR gene and its complex downstream signalling pathways [96]. Mutations in the coding region of the AR gene have been found in both untreated and hormone-refractory prostate cancer [97]. Segawa et al [98] demonstrated that AR expression was significantly lower in adenocarcinoma than is non-tumour prostate tissues. They also found that there is significant correlation between progression free survival and AR expression or proliferative activity. High AR expression predicted high proliferative activity and short progression free survival. Similarly, the results of Miyoshi et al [99] showed the AR expression level in hormone-refractory prostate cancer specimens was significantly lower than that in previously untreated prostate cancer or benign prostatic hyperplasia (BPH) specimens. The results suggested that a higher AR expression level result in poor recurrence-free survival and poor overall survival in hormone-refractory prostate cancer patients. The greater AR heterogeneity in poor responders may reflect a greater genetic instability in tumours that have progressed toward androgen independence i.e., many of the growth factors may exhibit their effects via crosstalk with AR [100]. AR heterogeneity may consequently be used as a predictor of treatment response and as a sign of progression [101]. Magi-Galluzzi et al [102] suggested that the heterogeneity in the expression of the androgen receptors increases with progression of invasive prostate cancer and might in part account for variable response to endocrine therapy.

#### Prostate specific antigen (PSA)

Prostate cancer causes the release of a number of substances into the blood stream. Of these, prostate specific acid phosphatase (PSAP) and prostate specific antigen (PSA) are clinically most important, and can be used for screening for prostate cancer, and for monitoring the response to treatment. To a degree PSA also provides diagnostic information [103]. Of the molecular forms of PSA, especially the free PSA seems to be useful for the detection of prostate cancer in men whose total PSA concentrations fall in the 4–10 microg/l range [104]. The values of total PSA (tPSA), free PSA (fPSA) and PSA complexed to alpha1-antitchymotrypsin (PSA-ACT) are all independent prognostic factors of prostate cancer survival [105]. Serum PSA levels are strong prognostic determinants of outcome following radiotherapy for prostate cancer and appear to add prognostic information independent of tumour stage and grade [106]. After radical prostatectomy a rising PSA level almost always precedes clinical recurrence of carcinoma [107]

The clinical significance of pre-treatment serum PSA values studied by Kuriyama et al [108] revealed that serum PSA can be used to predict the stage and prognosis of prostate cancer. Specifically, preoperative serum PSA levels are highly predictive of risk of recurrence after radical prostatectomy [109].

Determination of the serum urine PSA ratio enhances the specificity of PSA in screening, and in monitoring of patients with prostate cancer under androgen deprivation therapy [110]. An interesting study by D'Amico et al [111] revealed that men whose PSA level increases by more than 2.0 ng per millilitre during the year before the diagnosis of prostate cancer may have a relatively high risk of death of prostate cancer despite having undergone radical prostatectomy. Pre-treatment PSA level of 20 ng/ml or above, is of predictive value for the survival of men with clinically localized prostate cancer. Whether models based on pre-treatment PSA are as useful for predicting long-term survival as the other prognosticators like Gleason score, pathological lymph node status, and tumour stage will require further studies of longer follow-up to determine [112].

#### Microvessel density (MVD)

Tumour growth beyond a certain size requires angiogenesis. Once the tumour leaves the pre-angiogenic phenotype to become angiogenic, also metastases often start to evolve. Evaluation of the formation of new blood vessels has been proposed to provide important prognostic information in prostate cancer. The microvessel density count in the tumour area significantly increased with increasing Gleason score and nuclear grade. However, corresponding significant increase was not observed in the adjacent benign prostate or area of prostatic intraepithelial neoplasia (PIN) in the same samples [113]. Hall et al [114] concluded that quantification of tumour angiogenesis might prove valuable as a negative prognostic indicator in patients with localized prostate cancer. Borre et al [115] demonstrated that MVD was a significant predictor of shorter disease-specific survival in the entire cancer population as well as in the clinically localized cancer population. Halvorsen et al [116] concluded that in moderately differentiated prostatic adenocarcinoma MVD might improve the prognostic stratification of patients after radical prostatectomy. In contrast to these results, other studies [117,118], suggested that MVD (vessels marked by CD31) is not a useful prognostic indicator for men with clinically localized prostate cancer. Gettman et al [119] concluded that MVD assessed by both OMVD (optimized microvessel density) and AWMVD (area-weighted microvessel density) did not predict recurrence of pathologic stage T3 adenocarcinoma of prostate. Microvessel density does not seem to be a prognosticator of disease-specific survival as compared to conventional pathology combined with p53 and retinoblastoma assessment [120].

#### p53

Mutations of the p53 tumour suppressor gene can result in uninhibited cellular growth and have been implicated in numerous malignancies [121]. In most human cancers, increased immunohistochemical expression is associated with point mutations in one allele of p53 gene and loss in the other. Thomas et al [122] and Shurbaji et al [123] evaluated the immunohistochemical detection of p53 protein in prostate cancer and its utility as a prognostic indicator. They concluded that mutations of p53 gene, which have long half-life, are involved in carcinogenesis of prostate cancer, and that p53 reactivity marks an aggressive subset of prostate cancer. To compare potential biologic markers with laboratory, clinical and histopathologic parameters and PSA level, tumour stage, tumour grade, and DNA content, Papadopoulos et al [103] characterized the proliferation marker Ki-S5, p53 expression, and ploidy status as potential tumour biomarkers. High values of these markers in immunohistochemistry reflected poor prognosis.

Protein expression of p53, Ki-67, and bcl2 were evaluated in archival paraffin-embedded radical prostatectomy specimens from 162 patients of clinically localized cancer by Moul et al [124] to determine the clinical use of p53, Ki-67, and bcl2 immunohistochemical protein expression in the primary tumour as combined predictors of disease progression. The study concluded that p53, Ki-67, and bcl2 have potential as biomarkers to predict recurrence in patients with clinically localized prostate cancer after radical prostatectomy. All three markers were clearly correlated with recurrence estimates at 6 years. The same conclusion was obtained by Bauer et al [125].

Grignon et al [126] studied 471 patients to assess the prognostic value of identifying abnormal p53 protein expression in tumours of patients with locally advanced prostate cancer who were treated with either external-beam radiation therapy alone, or total androgen blockade before and during the radiation therapy. Statistically significant associations were found between the presence of abnormal p53 protein expression and increased incidence of distant metastases, decreased progression-free survival, and decreased overall survival. Among patients receiving both radiation therapy and hormone therapy, those with tumours exhibiting abnormal p53 protein expression experienced a reduced time to the development of distant metastases.

Seventy-one patients with clinically localized prostate cancer treated with radical prostatectomy were assessed by Theodorescu et al [127] to investigate whether the levels of immunoreactivity for p53, Rb, and bcl2 are better predictors of disease specific survival than conventional pathological parameters of the primary tumour, such as Gleason's score, capsular penetration, seminal vesicle invasion and percent of tumour in the specimen. They found that high level staining of p53 and Rb are independent factors predicting disease specific survival better than low level staining. They concluded that p53 and Rb immunohistochemical staining scores were superior to conventional pathological prognostic factors of the primary tumour as predictors of disease specific survival. In multivariate analysis, p53 overexpression was identified as the only prognostic parameter for recurrence-free survival (P = 0.005) [128]. Further prospective studies are recommended to confirm the independent prognostic potential of p53 overexpression in patients with localised prostate cancer, but it seems that p53 expression has the potential to become a dominant prognosticator in clinical practice.

Scherr et al [129] evaluated the expression of two key regulators of apoptosis, bcl2 and p53, by immunohistochemical staining on pre-treatment needle biopsies from 54 patients who were later treated with radiotherapy for localized prostate cancer. They found that biopsies with positive bcl2 and p53 expression were associated with treatment failure after external beam radiation therapy. These finding suggested that determination of bcl2 and p53 expression in pre-treatment stage may be helpful for predicting response to definitive radiotherapy.

#### p27

p27 is an inhibitor of the cell cycle with potential tumour suppressor function; it belongs to the Cip/Kip family of cyclin-dependent kinase inhibitory proteins which down regulate cell proliferation. Decreased levels of p27 protein expression have been correlated with poor prognosis in patients with breast [130,131], lung [132], and ovarian cancers [133].

To evaluate the prognostic value of p27 protein levels in patients with localized prostate cancer, Yang et al [134] examined 86 patients with clinical stage T1-2 prostate cancer who were treated with radical prostatectomy. The archival paraffin embedded specimens were sectioned and immunostained with p27 antibody, and scored by two independent observers in a blind fashion. They found that absent or low levels of p27 protein expression were an adverse prognostic factor in patients with clinically organ confined disease. This marker appears to be especially useful in patients with pathological stage T2-T3b disease.

#### p21

p21/WAF1 protein is a cyclin-dependent kinase inhibitor able to arrest the cell cycle at the G1 phase by inhibiting DNA replication. To understand the molecular mechanism leading to androgen-independent growth in prostate cancer, Baretton et al [135] demonstrated that p21/WAF1 overexpression before and after androgen deprivation therapy (ADT) characterized a subgroup of advanced prostate cancer with paradoxically high proliferation rate. Overexpressing cancers had significantly worse clinical outcome than cancers with low expression level.

Another type of p21 (ras p21) was studied by Agnantis et al [136]. They showed that ras p21 is detected in benign and cancerous lesions of the prostate gland. The expression tends to be weak in adenomatous hyperplasia and more intense in cancer. They found an inverse relation between ras p21 positivity and the degree of differentiation. The follow up of this study revealed a statistically significant correlation between 5-year survival and p21 expression, more intense staining reflecting poor survival.

### DNA ploidy

#### DNA content

The first report on the relationship of DNA ploidy of prostate carcinoma with prognosis appeared in 1966 [137]. It has been suggested that cytological smear preparations are more suitable than tissue sections for determination of DNA content and morphometric parameters such as nuclear shape, size, and texture due to less overlap between cells and between cell nuclei [138].

DNA studies have shown that patients with diploid cancers have longer disease-free interval and survival times than those with non-diploid tumours [139]. However, they may not be so helpful in predicting stage for an individual patient. In a multivariate analysis Forsslund et al [140] showed that DNA ploidy was a better predictor of survival than histological grade and tumour stage. Frankfurt and his colleagues [141] examined 45 patients with prostate cancer and noted that all 11 patients with organ confined cancer had diploid tumours. None of the aneuploid tumours were organ confined. Blute et al [142] selected two equal groups of age-matched patients for study. The patients were followed for 5–21 years with a median follow-up of 8 years. Both groups initially had clinical and pathologic stage B disease and were treated uniformly by radical surgery without adjuvant therapy. Members of one group had disease progression, whereas members of the other group did not. Although tumour grade, capsular involvement, the number of tumour foci, and tumour volume were evaluated in addition to DNA ploidy, disease progression showed a significant relationship with ploidy only. In the group with disease progression, 37% had diploid tumours, and 63% had non-diploid tumours (of which 34% tetraploid and 29% aneuploid). In contrast, the group without progression had 92% diploid tumours, and 8% non-diploid tumours (all tetraploid).

The most convincing evidence of the prognostic role of DNA content comes from a study by Forsslund and Zetterberg [143] where DNA was measured in a series of patients with a long-term follow-up. Patients who died within 3 years of diagnosis consistently had DNA stemlines at 3c and 6c, whereas long-term survivors (> 15 years) had stemlines at 2c and 4c. Stephenson et al [144] examined the ploidy value of cancer found in pelvic lymph node metastases and observed a strong correlation between mean survival time and ploidy status. In the Mayo Clinic prostatectomy series, ploidy was one of the significant predictive factors found in multivariate analysis of tumour characteristics [145].

The long-term outcome of 790 patients with adenocarcinoma of prostate treated with radical prostatectomy and early androgen ablation was assessed retrospectively by Seay and co-workers [146]. They found that diploid tumours were less likely to have biochemical (PSA test), local or systemic progression than non-diploid tumours. They concluded that patients with diploid tumours had a more favourable outcome than those with non-diploid tumours when treated with androgen ablation therapy. Ross et al [147] used needle biopsies for prediction of pathological stage and disease recurrence after prostatectomy by DNA ploidy analysis. The study showed a ten-fold increase in risk for metastasis and three-fold risk for extra-capsular spread if the initial needle biopsy demonstrated non-diploidy. The authors concluded that ploidy analysis was more important than grade in prediction of outcome.

In a study of stage C prostate cancer, Lee S et al [148] found that there was a greater likelihood for recurrent disease after surgery if tumours were non-diploid. The probability of a disease-free interval of 60 months was 85% for those with diploid tumours as compared with only 9% for those with non-diploid tumours. This advantage held even in the presence of seminal vesicle involvement: 73% of patients with diploid tumours and seminal vesicle involvement remained free of disease during follow-up, as compared with only 8% of those who had non-diploid cancer and seminal vesicle involvement.

Buhmeida et al [149] studied the influences of sampling rules on the appearance of histograms in fine needle aspiration biopsies of prostate. He found that diploid or peridiploid patterns were commonly found in cell groups while tetraploid, peritetraploid, or aneuploid patterns were clearly more common among free cells. The results strongly suggested that measurements on cell groups were less efficient in detecting abnormality than the measurements done on free cells. The clinical relevance of the histogram patterns were also examined [150] with respect to their significance in differentiating between benign and malignant cells and also in estimating the stable or progressive character of prostate cancer. The aneuploidy patterns and DNA histogram characteristics defined by the presence of cells above cut-off points of > 5c, > 6c, > 7c and > 8c seem to be useful in differentiation between the stable and progressive characteristics of cancer, i.e. between the more aggressive types of cancer and cancers with slower progressive activity.

### Cell proliferation

#### Ki-67

Ki-67 is one of several cell-cycle-regulating proteins, which can be demonstrated by immunohistochemistry [151,152]. It is a DNA-binding protein, which is expressed in all phases of cell cycle but undetectable in resting cells [153,154]. Ki-67 index (fraction of Ki-67 positive nuclei in immunohistochemistry) was higher for carcinomas than for hyperplastic glands. Within the group of carcinomas, Ki-67 indices in patients with metastatic disease were significantly higher than in those without metastasis. However, the results suggested that high Ki-67 index could define a group of patients with poor prognosis [155].

To identify associations between proliferative indexes (including Ki-67 index) and disease progression following radical prostatectomy, paraffin embedded specimens from 180 patients were immunohistochemically stained for Ki-67 antigen by Bettencourt and his colleagues [156]. They found that patients with a high Ki-67 antigen score had earlier progression and a lower 5-year recurrence-free survival rate than those with low or negative Ki-67 antigen score (p < 0.001). The same was concluded by Bubendorf et al [157]. The results of a study by Aaltomaa et al [158] showed that Ki-67 expression is a potentially useful predictor of survival (p = 0.025) in prostatic adenocarcinoma. In contrast to these studies, Ojea Calvo et al [159] suggested that Ki-67 expression (3%) in preoperative biopsies is less effective than classic factors such as PSA, Gleason score, and pT classification in predicting prostate cancer biochemical progression after radical prostatectomy.

#### S-phase fraction

S-phase fraction (SPF) is the proportion of cells in the S phase of the cell cycle. S-phase fraction can be estimated from DNA flow cytometry (FC) histograms and also from DNA static cytometry histograms [160]. The results based on these two methods to evaluate proliferation parameters generally give similar results [161,162]. Although that some differences may exist. High SPF is associated with shorter overall survival and shorter time to local progression and metastasis in clinically localized prostate cancer [163]. Bratt et al [164] compared the prognostic significance of S-phase fraction with chromosome aberrations and DNA ploidy in prostate adenocarcinoma. They found that SPF as determined with FC was superior to karyotype and ploidy in predicting poor survival in prostate cancer.

#### Gene expression profiling

DNA microarray technology is revolutionizing the way fundamental biologic questions are addressed in the postgenomic era. Microarrays allow a simultaneous gene expression analysis of thousands of genes, providing an expression profile of the specimen investigated. The idea is to find gene expression profiles associated with good or poor clinical outcome in terms of survival or in terms of treatment response in prostate cancer [165,166]. The progress in this field has been shown particularly in breast cancer [167,168]. The use of a microarray based prognostic tool in prostate cancer is under development. However, some studies have implemented high-density DNA microarrays to analyze prostate cancer specimens [169-171]. Gene expression profiles of invasive and organ-confined prostate cancer have been established [172], as these have been correlated with clinical behaviour [173].

## Conclusion

The majority of prostate carcinomas never progress to clinically significant disease. A minor fraction of the clinical cases remains confined to the prostate for many years and other carcinomas progress rapidly to a life threatening disease. How to distinguish these three biologically different types [174], is a question of great importance. Pathologists play an important role in preoperative diagnosis and in the postoperative prognostic evaluation. Most research results currently available were based on radical prostatectomy specimens to find markers on the basis of disease progression could be predicted. This means that they need to be tested on biopsy material before the treatment decision to be taken. But the multifocal and heterogeneous nature of prostate cancer makes it difficult to obtain a representative biopsy sample. Improvements in biopsy procedures will be mandatory in order to make progress on this issue [175,176].

Histological grading is a very important factor for the assessment of prognosis. Although the reproducibility is not perfect, still the Gleason's grading system is the most favoured prognostic factor, and highly significantly associated with survival and/or progression. Additionally, the volume of the tumour, vascular invasion, extension of the tumour through the prostate capsule, and invasion to the seminal vesicle might be valid prognostic factors for disease progression and survival. The value of different biomarkers (p53, ki-67, androgen receptor mutations, IGF, E-cadherin) remains to be applied in clinical practice [171,177,178].

DNA ploidy is a good prognostic factor after prostatectomy and can be used in planning therapy. Unfortunately DNA ploidy measurements from biopsies are rare in clinical practice, in spite of the extensive literature that supports their use [173]. Compound prognostic factors based on the gene expression profiles (tested by DNA arrays) are promising and will accelerate the discovery of new predictive and prognostic molecules, but clinically relevant data up to this moment are still lacking [179]. Multivariate analyses of prognostic factors are enough, and multivariate models for prediction of compound prognosticators or predictors have not been well tested in clinical practice.

Approximately one third of clinically localized prostate cancer treated by radical prostatectomy will recur within 10 years. To prevent recurrence, new adjuvant therapies are in development for treating high-risk patients after surgery [180-182]. To identify good candidates for these treatments, there is still a need for new biomarkers that potentially will improve the ability of evaluation of the prognostic or predictive status of the patient [183].
